# Legal Disputes over Duties to Disclose Treatment Risks to Patients: A Review of Negligence Claims and Complaints in Australia

**DOI:** 10.1371/journal.pmed.1001283

**Published:** 2012-08-07

**Authors:** Marie M. Bismark, Andrew J. Gogos, Richard B. Clark, Russell L. Gruen, Atul A. Gawande, David M. Studdert

**Affiliations:** 1 From the Melbourne School of Population Health, University of Melbourne, Carlton, Victoria, Australia; 2 Department of Surgery, Royal Melbourne Hospital, Parkville, Victoria, Australia; 3 National Trauma Research Institute and The Alfred, Melbourne, Victoria, Australia; 4 Harvard School of Public Health, Boston, Massachusetts, United States of America; 5 Melbourne Law School, University of Melbourne, Carlton, Victoria, Australia

## Abstract

David Studdert and colleagues identified disputes over informed consent among malpractice claims and serious health care complaints in Australia and provide an analysis of disagreements between patients and doctors over whether particular clinical risks should have been disclosed before treatment.

**Summary Points**

- Doctors, especially surgeons, are often unsure which clinical risks they should disclose and discuss with patients before treatment. Leading medical malpractice cases in many countries have centered on this issue.
- In a sample of nearly 10,000 malpractice claims and conciliated health care complaints from Australia, we identified 481 disputes over informed consent, 45 (9%) of which were “disputed duty cases”—disagreements between patients and doctors over whether a particular clinical risk should have been disclosed before treatment.
- Two-thirds of disputed duty cases involved surgical procedures, and the majority (38/45) of cases related to five adverse outcomes: the need for further surgery, poor cosmetic result, impaired vision or hearing, chronic pain, and infertility or sexual dysfunction.
- The most common justifications doctors gave for non-disclosure were that the risk was too rare to warrant discussion or the specific risk was covered by a more general risk that was discussed.
- Although most informed consent disputes appear to involve disagreements about who said what and when, not stand-offs over whether a particular risk ought to have been disclosed, doctors may routinely underestimate the importance of a small set of risks that vex patients.

## Introduction

The 1972 case of *Canterbury v Spence* [1] ranks among the best-known court decisions in American and international health law. Mr Canterbury, a 19-year-old typist for the Federal Bureau of Investigation, became paraplegic and incontinent following spinal surgery. He sued, alleging that the surgeon, Dr Spence, had failed in his duty to outline the risks of this outcome. Dr Spence countered that he owed no duty to warn of such an unexpected complication. The enduring significance of the case lies in the decision by the District of Columbia Court of Appeals to reject the traditional customary standard for assessing negligence (*what would a reasonable practitioner have done?*), and opt instead for a new patient-centered standard (*what would a reasonable patient want to know?*).

In the 40 years since *Canterbury*, appellate courts of many US states [2] and many countries—including the United Kingdom [3], Canada [4], Australia [5], Malaysia [6], Ireland [7], and New Zealand [8]—have considered similar cases, disputes in which patients and doctors square off over whether a particular treatment risk ought to have been disclosed. (Descriptions of these cases are provided in Table S1.) Many jurisdictions have moved toward legal standards for risk disclosure that prioritise patient preferences. This general shift compounds an uncertainty that doctors, especially surgeons, regularly face: which types of risks should be emphasized in the consent process?

While the duty to disclose risks has been analysed and critiqued extensively in the health law and bioethics literature [9], this scholarship is largely normative [10]. Remarkably little is known about the clinical circumstances in which doctors and patients disagree about whether a particular risk ought to have been disclosed (“disputed duty cases”).

We identified 481 legal disputes over informed consent to medical treatment in Australia. The disputes were drawn systematically from litigation and conciliation files resolved over a seven-year period. In a recent report [11] we described general characteristics of this sample. In this analysis, we describe a subset that involved explicit disagreements between patients and doctors about whether a particular risk ought to have been disclosed. Our aim was to detail the treatments, risks, and adverse outcomes at issue in these cases.

## Analysis

### Setting

Avant Mutual Group Limited (Avant) and the Office of the Health Services Commissioner of Victoria (HSC) provided data for our anlaysis. Avant is Australia's largest provider of medical indemnity insurance, covering approximately 55% of the country's registered medical practitioners. The HSC, established in 1987, has statutory responsibility for resolving complaints against health care providers in Victoria, Australia's second most populous state with 5.2 million residents. Patients must initiate complaints in writing but do not require legal representation. The system is free and open to all and is advertised widely in health care facilities.

### Data

The sample frame consisted of all malpractice claims (*n* = 7,846) brought against doctors insured by Avant in three states (New South Wales, Victoria, and Queensland) between 1 January 2002 and 31 December 2008, and all conciliated complaints (*n* = 1,898) lodged with the HSC in Victoria during the same period. (The HSC data related to all complaints against doctors, regardless of who insured them.) We have previously described our method for screening claims and complaints in this sample frame to determine which ones met the definition of an informed consent dispute [11]. We recap definitions of key terms in Box 1.

**Box 1. Key Definitions**

A ***claim*** is a written demand for compensation.

A ***conciliated complaint*** is a complaint the HSC considers too complex or serious to be resolved through facilitated communication alone, and so refers it to formal conciliation. (Approximately 20% of all complaints lodged with the HSC proceed to conciliation.)

An ***informed consent dispute*** is a claim or complaint that alleges a deficiency, either in the quality or quantity of information provided to the patient about a treatment prior to a decision about whether to undertake it, or in the process through which the patient was asked to consider such information and make a decision.

***A disputed duty case*** is a type of informed consent dispute, one that involves a head-to-head disagreement between a patient and a doctor over the need to explain certain risks. These are situations in which a patient (or the patient's representative) alleges that a particular risk should have been disclosed before treatment, and a doctor responds that the disclosure was unnecessary or inappropriate.

Data collection proceeded in two steps. We first undertook an initial review of cases in the parent study [11] and then followed up with an in-depth review reported here. In the follow-up review, one investigator (MMB) returned to Avant and HSC offices between August and November 2010 and re-examined the hardcopy files associated with all cases flagged in the initial review as meeting the study definition of a disputed duty case. We confirmed that the cases met the study definition and collected supplementary information, including details of patients' allegations and health outcomes, doctors' responses, and the undisclosed risks in dispute. Probabilities associated with the clinical risks in selected cases were subsequently obtained through a series of Medline searches and literature reviews (one per case).

We did not attempt to judge whether the patient's or doctor's position in the disputed duty cases was the correct one. Doing so would have required more information than was available to us in the case files. The case outcomes are not an appropriate proxy for merit. With claims, cases were typically resolved by out-of-court negotiation. Moreover, allegations about deficiencies in the consent process often co-existed with other types of allegations, yet legal outcomes were generally “global”, not tethered to specific allegations. With complaints, the HSC runs a dispute resolution process; it generally does not rule on the merit of patients' allegations or practitioners' responses.

The ethics committee at the University of Melbourne approved the study.

## Findings

### Frequency of Disputed Duty Cases

**Figure 1:**
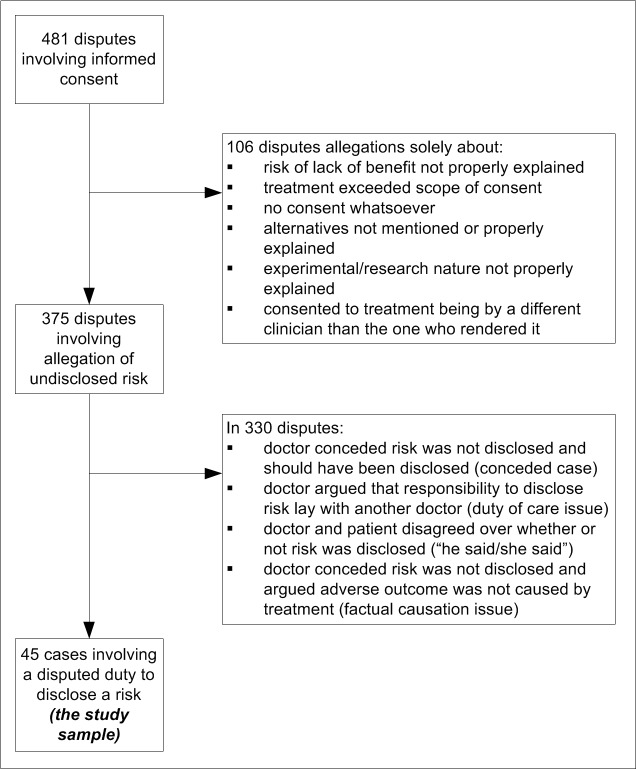
Derivation of study sample.

A total of 3.4% (263/7,846) of malpractice claims and 11.5% (218/1,898) of conciliated complaints involved disputes over informed consent. Three-quarters (375/481) of informed consent disputes involved allegations that risks had not been disclosed (Figure 1). However, most of these cases (88%, 330/375) were not disputed duty cases because they did not involve disagreements between patients and clinicians over whether a risk ought to have been disclosed.

Rather, factual disagreements predominated. These were chiefly factual disputes about whether the risk had been disclosed before treatment (e.g., “I would have discussed the risks of thrombosis associated with this contraceptive”) or whether the patient's poor outcome was due to materialisation of the undisclosed risk (e.g., “There was no causal connection between the iodine discogram and her thyroid disease”). In addition, in several cases the doctor conceded that the risk was not disclosed but should have been (e.g., “I didn't disclose the risk of bile duct injury. I apologise for this and think the case should be settled.”).

Nine percent (45/481) of informed consent cases were disputed duty cases. All findings reported hereafter pertain to this special group of cases.

### Treatments and Adverse Outcomes

In more than two-thirds of disputed duty cases, the treatment rendered was a surgical procedure (31/45). The rest involved medications (7), anaesthetic procedures (3), obstetric care (3), and a washout of tear ducts performed by a general practitioner.

Table 1 shows the types of adverse outcomes for patients that resulted from materialisation of the undisclosed risks. In a third of cases (15/45), patients complained of not being warned of the risk that further surgery would be needed; in nearly three-quarters of cases (33/45) the complaint centered on not being warned about one of four outcomes: chronic pain, impaired vision or hearing, poor cosmetic result, and infertility or sexual dysfunction.

**Table 1:** Physical health outcomes associated with undisclosed risks that materialised in disputed duty cases.

Outcomes	*n* (%)^a^
Further surgery required	15 (33%)
Chronic pain	13 (29%)
Poor cosmetic result or delayed wound healing	9 (20%)
Impaired vision or hearing	8 (18%)
Infertility or sexual dysfunction	7 (16%)
Paralysis	3 (7%)
Other	12 (27%)

a Total sums to greater than 45 because categories are not mutually exclusive.

The dominance of these five outcomes among disputed duty cases is striking: collectively, they featured in 84% of disputed duty cases. (It is also worth noting that several of the leading court cases, detailed in Table S1, involved this same group of outcomes.) What these outcomes have in common is important quality-of-life implications for patients. Our findings suggest that doctors may underestimate the premium patients place on understanding the risks of them in advance of treatment.

The adverse outcomes enumerated in Table 1 are essentially physical in nature. Patients in approximately a third of cases (17/45) also alleged psychological harm, in the form of depression or an anxiety disorder, associated with the adverse outcome.

### Doctors' Justifications for Non-Disclosure

Table 2 shows the distribution of cases by type of justification doctors gave for not having disclosed the risk. Examples of selected cases are also shown. The “risk too rare” and “subset of general risk” justifications for non-disclosure were particularly common; collectively, they appeared in nearly two-thirds of the disputed duty cases.

**Table 2:** Doctors' justifications for non-disclosure of risk in disputed duty cases.

Justification Type	*n* (%)	Case Examples	
		Treatment and Health Outcome	Excerpts from Doctors' Justificatory Statements
Rare risk	18 (40%)	Cyclosporin leading to tinnitus and hearing lossRectal prolapse repair leading to inability to ejaculate	“It is not our practice to mention all rarely reported side-effects of every medication that is prescribed.”“I would not specifically have warned of potential sexual difficulties because the incidence should be relatively low.”
Subset of general risk	11 (24%)	Migration of gastric reflux collar leading to cardiac tamponadePhenytoin leading to Stevens-Johnson syndrome causing blindness	“I had mentioned Angelchik collars had been known to migrate. I had certainly not mentioned this exceedingly rare complication.”“I did not warn him specifically of Stevens Johnson syndrome though I did discuss allergic reactions in general.”
Obvious or implied risk	5 (11%)	Cosmetic eyelid surgery leading to post-operative infection	“Although I may not have highlighted problems of infection, most people, particularly if they are married to a doctor, would be aware that any operation can be complicated by infection.”
Duration of risk	4 (9%)	Cosmetic breast surgery with poor wound healing after one year	“Most would be healed within a couple of months. The maximum time I would have expected would be six months.”
Risk clearly outweighed by benefits	4 (9%)	Abdominal lipectomy leading to post-operative infection and scarring	“Infection is a solvable problem and the patient would still be in a better position than before the surgery.”
Risk of negligence	3 (7%)	Gastric lap band leading to perforation of right ventricle by liver retractor	“I did not mention specifically perforation of the heart, but this could be understood as this has never been reported before.”

#### Risk too rare

The most common justification for non-disclosure (18/45 cases) was that the risk was too rare. These were cases in which doctors argued that the outcome the patient experienced occurs too infrequently in clinical practice to warrant disclosing it during the informed consent process, or the risk was so rare that it was unknown to the doctor.

#### General risk was disclosed

The next most common justification (11/45 cases) was that the risk not discussed was encapsulated in a general risk that was discussed. In a quarter of cases, for example, the doctor mentioned generic risks such as bleeding or infection without providing specific information regarding possible consequences for the patient. In another case, a doctor had warned the patient of the risk of an allergic reaction to phenytoin, but had not specifically mentioned the risk of Stevens-Johnson syndrome and blindness. These findings are consistent with research suggesting that clinicians tend to be overly general in their descriptions of some risks, and struggle with discussing serious complications in specific terms [12].

#### Other justifications

Each of the other types of justification applied to relatively few cases. Doctors defended non-disclosure in five cases by arguing that the risk was obvious and a reasonable patient ought to have been aware of it. In four cases, the doctor argued it was sufficient to have advised the patient of the average recovery time for a procedure and been silent on risks of delayed recovery; all of these cases involved cosmetic procedures.

Doctors in a further four cases argued that the need for disclosure was obviated by the fact that the benefits of the treatment clearly outweighed any risks, and disclosing the risk in question would have imposed an unnecessary burden on the patient; all of these cases involved surgical procedures. Arguments that it is unnecessary or inappropriate to “burden” the patient with information about procedures they are about to undergo are paternalistic; they hark back to an earlier era in which there was greater deference to the medical profession, and such exercises of “therapeutic privilege” were common and accepted [13],[14].

The final three cases were unusual in that the adverse outcome was patently due to negligent care. The patients in these cases alleged a failure to warn of the risk of the outcome and the doctors argued the risk was not one they needed to disclose. (Technically, the doctors were probably correct, because there is no legal duty to warn of risks arising from negligent care.)

### Rare Risk Cases

Table 3 provides details of the treatments, adverse outcomes, and risk probabilities for the 18 disputed duty cases in which the doctors' justification for non-disclosure was that the risk was too rare to warrant it. Our literature review indicated a wide span in these probabilities, ranging from complications described in only a few case reports (e.g., testicular loss following varicocoele repair) to well-recognised adverse outcomes occurring in over 1% of cases (e.g., fetal laceration during caesarean delivery of a breech baby).

**Table 3:** Characteristics of 18 disputed duty cases in which doctors' justification for non-disclosure was risk too rare.

Treatment	Risk That Materialised	Probability of Risk
Topical steroid for eczema	Steroid induced rosacea	>1% [23]
Rectal prolapse repair	Inability to ejaculate	>1% [24]
Caesarean for breech baby	Fetal laceration requiring surgical repair	>1% [25],[26]
Prolonged prednisone for erythematous eruptions	Avascular necrosis requiring hip replacement	>1% [27]
Laparoscopy for endometriosis	Removal of ovary and tube to control bleeding	0.1 to 1% [28]
Cyclosporin for psoriasis	Tinnitus and hearing loss	0.1 to 1% [29]
Inguinal hernia repair	Testicular necrosis requiring orchidectomy	0.1 to 1% [30]
Spinal anaesthetic for caesarean	Paraesthesia and weakness in leg	0.01 to 0.1% [31]
Laryngeal mask airway	Loose teeth requiring dental surgery	0.01 to 0.1% [32],[33]
Laparoscopic gastric banding	Cuff leak after 15 months requiring surgery to replace band	0.01 to 0.1% [34]
Flucloxacillin	Hepatitis	<0.01% [35]
Bilateral inguinal hernia repair	Infertility due to azoospermia	<0.01% [36],[37]
Vaginal delivery of 4.5 kg baby	Diastasis of symphysis pubis	<0.01% [38]
Varicocoele repair	Testicular infarct requiring orchidectomy	Case reports [39]
Vasectomy	Sperm granuloma requiring surgical excision	Case reports [40]
Coronary angiogram and angioplasty	Damage to aortic valve requiring emergency cardiac surgery	Not available
Dilatation/washout of tear ducts	Lacrimal duct stenosis leading to inflammation and impaired vision	Not available
Pelvic osteotomy for scoliosis	Cosmetic deformity (prominent lateral hip)	Not available

There was no obvious pattern to this wide variability. We had expected an inverse correlation between risk frequency and severity in this group of cases, but found no evidence of one. Nor was there evidence of convergence on a standard risk threshold: the probabilities appeared to vary across several orders of magnitude, from less than 0.01% to greater than 1%.

## Discussion

### Disputed Duty Cases in Context

Landmark court battles [1],[3],[4],[5],[6],[7],[8] over informed consent have centred on what legal standard of care should apply in head-to-head disputes between patients and doctors over whether a treatment risk warrants disclosure. To the best of our knowledge, this is the first study to examine this type disagreement at the population level. The finding that nine out of ten legal disputes over informed consent cases did not turn on such a disagreement highlights a general point: highly publicized legal cases—those at the apex of the “dispute pyramid”—can easily distort understanding of the much larger number of “garden variety” disputes and grievances that sit beneath them [15],[16].

The finding also has a practical message for practicing clinicians: malpractice claims and complaints over informed consent are not uncommon events, but when they arise they are most likely to centre on mundane factual disagreement over who said what and when, not contests over what *should* have been disclosed. This underscores that for the informed consent process, like most other areas of clinical practice, regular and careful documentation of interactions with patients is a prudent risk-management strategy. Documentation of the details of consent discussions in the lead-up to surgical procedures is particularly important, as the vast majority of informed consent disputes involve complications following operations [2],[11].

Despite their rarity, disputed duty cases are of special interest for several reasons. From a legal standpoint, these are the types of cases that define and test the standard of care to which doctors must adhere in obtaining informed consent. From a medical standpoint, the clinical details of disputed duty cases may point to an important “penumbra” of treatment risks—outcomes about which there is division or uncertainty among doctors as to the appropriateness of disclosure and warning. From a patient standpoint, disputed duty cases may highlight certain types of risks that patients tend to prioritise more highly than doctors do. What lessons does an analysis of such cases have for how doctors should approach the informed consent process?

## What to Disclose: A Balancing Act

The clinical reality is that standardised consent forms are widely used, particularly for common procedures, and they tend to present exhaustive enumerations of risks. Anglo-American courts do not accept that merely handing such forms to patients as a valid way to obtain informed consent. Consequently, clinicians must determine which risks to discuss and emphasise. For busy doctors this necessitates choices because time is limited and effort devoted to consent discussions has opportunity costs [17].

One approach is to focus discussion on risks of outcomes above a certain incidence. The notion of a 1% risk threshold appears to have some currency in clinical practice. However, it has no firm basis in either law or available evidence regarding patients' attitudes to risk [18],[19],[20]. Courts regard the probability of a particular adverse outcome as an important element in determining what qualifies as a “material” risk that must be disclosed, but it is one of several elements.

The severity of the outcome associated with a risk also matters. It is reasonable to think of rarity and severity as considerations that operate in tandem, on a sliding scale. Small risks of catastrophic outcomes usually warrant emphasis, as do high risks of relatively minor adverse outcomes, but not low risks of minor outcomes.

Distinctive characteristics of individual patients may also dictate the breadth and depth of discussion about certain risks; the extreme example of a hand operation on a concert pianist helps to illustrate the point. A less obvious consideration is the treatment's urgency. Details of risks tend to matter more toward the elective end of the treatment spectrum than the urgent or emergent end, which may help to explain the prominence of cosmetic treatments among the disputed duty cases in our sample.

To this recognised set of factors, our analysis draws attention to five outcomes that appear to trigger the majority of disputed duty cases—the need for further surgery, poor cosmesis, impaired vision or hearing, chronic pain, and infertility or sexual dysfunction. These are outcomes that clinicians may give too little weight and attention in the consent process.

## Limitations

Our analysis has several limitations. First, we examined legal disputes over the duty to disclose certain risks; this sample of cases may be unrepresentative of wider disagreements between patients and doctors in this area because they are refracted through the lens of patients' claiming and complaining behaviour [21]. Second, we were constrained by the information set available in claim and complaint files. Finally, the generalisability of our findings may be influenced by differences in medico-legal systems and, in particular, the prevailing legal standard for informed consent. Since 1992, Australian courts have applied a patient-centred standard [5],[22]; the same standard prevails in around half of the states in the US [2] and in a number of other countries [4],[6],[7],[8], where the decision in *Canterbury v Spence* has proved to be influential.

## Conclusion

The rationale for informed consent springs from the ethical principle of autonomy—the notion that it is patients themselves who should make the final decision about which course of treatment to follow. Increasingly, doctors are expected to advise and empower patients to make rational choices by sharing information that may bear upon the decision, including risks of undesired outcomes. Occasionally, doctors and patients will disagree about whether a particular risk has an important bearing on treatment choices. Improved understanding of these situations helps to spotlight gaps between what patients want to hear and what doctors perceive patients want (or should want) to hear. It may also be useful information for doctors eager to avoid medico-legal disputes.

## Supporting Information

Table S1 **Leading court cases on informed consent from 7 countries.**
(DOC)

## Abbreviations

HSC: Health Services Commissioner of Victoria
