# Supplementary material for: Legal Disputes over Duties to Disclose Treatment Risks to Patients: A Review of Negligence Claims and Complaints in Australia
Source: PLoS Med. 2012 Aug 7;9(8):e1001283. doi: 10.1371/journal.pmed.1001283 (PMC3413715; doi:10.1371/journal.pmed.1001283)
Supplement: Table S1 — Leading court cases on informed consent from 7 countries. (DOC) [file pmed.1001283.s001.doc]

**Table S1. Leading court cases on informed consent from 7 countries**

| **Case** | **Treatment** | **Undisclosed risk that materialised** |
| --- | --- | --- |
| *Canterbury v Spence*  United States (1972) | Laminectomy for a suspected ruptured disc | Paraplegia and incontinence |
| *Reibl v Hughes*  Canada (1980) | Carotid artery surgery | Stroke resulting in paralysis and impotence |
| *Sidaway v Bethlem Royal Hospital Governors*  United Kingdom (1984) | Laminectomy for shoulder and arm pain | Severe spinal cord damage resulting in partial paralysis |
| *Rogers v Whittaker*  Australia (1992) | Surgery on near-blind right eye | Sympathetic ophthalmia resulting in blindness in left eye |
| *Hong Chuan Lay v Dr Eddie Soo Fook Mun*  Malaysia (1998) | Surgery on cervical spine | Paralysis and incontinence. |
| *Geoghegan v Harris*  Ireland (2000) | Bone graft from chin for dental implant | Chronic neuropathic pain |
| *Harman v Director of Proceedings*  New Zealand (2009) | Breast reduction and abdominoplasty | Loss of nipple and scarring |
